# A Lecture on the Diseases of the Inferior Maxilla

**Published:** 1851-04

**Authors:** John Simon

**Affiliations:** Surgeon to St. Thomas' Hospital.


### ARTICLE XVII.

*A Lecture on the Diseases of the Inferior Maxilla.* Delivered by John Simon, Esq., Surgeon to St. Thomas' Hospital.

Gentlemen :—As the subject on which I mean to lecture to-day has not yet passed into the text-books of surgery, I shall tell you something of its history, before I detail the cases which will serve for its illustration.

During the last five years, several successive papers, by various German and French surgeons, have concurred to establish the fact, that persons exposed to the vapors of phosphorus—

as, for instance, in the manufacture of lucifer-matches, are liable to contract a disease, under which more or less of their jaws becomes necrosed, and exfoliates. Lorinser, a hospital-surgeon of Vienna, was the first who published anything definite on the matter; his paper bears date early in 1845, and the cases recorded in it run back to 1839. Shortly afterwards, Professor Heyfelder, of Erlangen, and M. Strohl, of Strasburg, furnished additional evidence, founded on their own independent observations; and since that time, a number of practical surgeons, here and elsewhere, have had occasion to note a repetition of the phenomena to which those original observers first drew attention.

In 1847, two Nuremburg professors, Von Bibra and Geist, (the one a chemist and physiologist, the other a surgeon,) published conjointly a monograph on the subject, which, in addition to a lucid summary of all that had previously been written, contains many valuable original statements of the anatomical and chemical changes observed in the affected bone. To those of you who read German, I would recommend the perusal of this book, not only as a compendium of all that is known on the subject, but likewise as an admirable instance of exact pathological investigation. Those who are unable to read it in the original language, may find a good summary of its contents in the second number of the *British and Foreign Medico-Chirurgical Review.* You will likewise find the subject alluded to in Mr. Stanley's recent work on the Diseases of the Bones.

Now, gentlemen, I may tell you, very briefly, the gist of what our foreign fellow-laborers have ascertained for us in respect of this new disease. Commencing with the general statement, I repeat that destructive disease of the jaw has been found frequently to occur among lucifer-match-makers; and the object of inquiry has been, starting from that vague supposed connection, to ascertain whether the relation between the employment and the disease be a real relation of cause and effect, and if so, what is the *modus operandi* of the phosphoric fumes, and what are the morbid processes which constitute the disease.

First, the reality of the connection has been established by experiments performed on the lower animals; the disease has been produced artificially in rabbits, by exposure to the phosphoric fumes, under circumstances similar to those which determine the disease in man. These circumstances require a little explanation. Hundreds of persons are occupied in lucifer-making, who sustain no injury from the process; a few, on the other hand, contract the disease with extraordinary readiness. Why do so few persons suffer? and why do they suffer always in the jaws?

To take the latter question first, you may be disposed to answer, from analogy, "that it is no uncommon thing for a poison to select a single spot for the local manifestation of its constitutional influence: that if phosphorus impregnates the blood injuriously, *some* organ is sure to be selected for its future destructive operation; and if some organ, why not the jaws? just as arsenic selects the stomach, mercury the gums, cantharides the kidneys, opium the brain; or just as the poison of secondary syphilis, when it attacks the bones, always settles preferentially on certain parts of the skeleton—on the tibia, the cranium, the clavicle," &c. And if it were suggested to you, instead of this explanation, that the jaws perhaps may derive their greater liability to the disease from their more immediate exposure to the fumes, and that, according to this view, the affection may be merely a local one, arising where the operation of the cause is most direct and most powerful; if, I say, this suggestion were made to you, you might object, (as Mr. Stanley does,) that "the vapor is also directly inhaled through the nasal passages, and yet it produces no effect on the bones which bound those passages, though they, equally with the jaws, are exposed to its influence as a local excitant."

Notwithstanding this apparent difficulty, the fact is, that the phosphoric fumes do operate only locally, and select the jaw only because of its greater exposure to the irritant. The two difficulties of explanation that I have referred to—first, that the maxillary bones are affected rather than the bones of the nasal cavities; and secondly, that the proportion of sufferers by the disease is very small in comparison with the large number of persons engaged in the manufactories: both these difficulties vanish with what I have now to tell you. The disease only attacks persons with carious teeth, and it operates in the buccal rather than in the nasal cavities, because it is only in the former that the phosphoric vapor is enabled, by carious teeth and ulcerated gums, to come into direct contact with the periosteum, on which its irritating influence is subsequently exerted.

In the experiments which Professor Von Bibra performed on rabbits, he found that the characteristic disease could only be produced when he had previously, by artificial means, denuded the periosteum of the jaw in the animals which he was about to expose to the chronic influence of the phosphoric atmosphere; and the persons in the lucifer manufactories who pursue the occupation with impunity, are those whose teeth are free from caries. Such persons suffer neither in the jaw nor constitutionally; contrary to what one might expect, the vapor appears incapable of producing any general cachexia, and the the utmost evil ascribed to its operation in the lucifer-works, beyond the one under consideration, seems to be that of irritating the respiratory passages and producing chronic bronchitis. This effect, however, appears to be unusual.

It appears, then, that the fumes of phosphorus, as evolved in lucifer-match manufactories, coming into immediate relation with the periosteum, are capable of producing, and ordinarily do produce, the disease in question; and it is suggested that, in all probability, the noxious agent is not phosphoric acid, since, in phosphorus manufactories, where the air is saturated with that acid, no such results are known. Probably, (it is inferred,) the agent is either some lower oxydation of phosphorus—perhaps, hypo-phosphorous acid, or else that mysterious phenomenon, ozone, which is said to be largely developed during the volatilization of phosphorus. To us, (as surgeons,) the determination of this question matters little. Whether the agent be an acid, soluble in the saliva, and capable of hastening the disintegration of the earthy substance of the jaw—

an hypothesis which to me, I confess, seems hardly tenable—or, (according to the other supposition,) be a principle which would exceedingly accelerate the oxydation and decay of the animal substance; in either case, for our purposes, we have a sufficient explanation of what ensues. For it is an axiom in pathology, that wherever you have an accelerated waste of tissue, *there* also you have an increased determination of blood; and I have had occasion, over and over again, to show you how easily the limits are passed which divide this merely *hypertrophic hyperæmia* from the phenomena and tendencies of active inflammation.

In the case of Brewerton, which I shall read presently, you will have an account of the ordinary symptoms which attend the disease; but I dare say it will be the more intelligible for my telling you first, generally, what are the processes to be observed.

The disease consists essentially in inflammation of the periosteum, terminating in necrosis or death of the bone. There is a first stage, in which the disease of the periosteum is akin to hypertrophy—a stage of increased vascularity and exalted function. During this stage, which is almost indefinitely chronic, the congested periosteum becomes infiltrated, of course, with more than the ordinary exudation of nutritive lymph; and this lymph, according to the normal function of the part, tends to develop itself into bone. Thus, in the lower jaw, at least, we have a laminated deposit of new bone, (possessed of certain microscopical and chemical peculiarities, on which I need not now insist,) taking place around the old one. Just as an inflammatory effusion in the belly gravitates to the pelvis, so, about the lower jaw, we find that this infiltration settles down towards the base; and consequently, that the largest amount of osteophytic incrustation lies about the inferior circumference of the bone. There is a remarkable difference, in this respect, between the two jaws. The greater porosity of the upper jaw probably invites more interstitial deposit into its structure than would be possible in the denser substance of the lower; and the remainder of the effusion from the inflamed periosteum seems, in this case, to find its way into the mouth—perhaps by the same gravitation which determines its subsidence *from* the mouth, where the lower jaw is affected. Be this as it may, the fact is, that the upper jaw does not show those osseous incrustations which are invariable in the lower.

A slow, periosteal hypertrophy, with gradual alteration of tissue, and, (in the lower jaw at least,) a laminated growth of bone in the periosteum and beneath it, constitute the first stage of the disease. This stage is accompanied by little inconvenience; and as the patient has carious teeth, whatever occasional flying pains are felt, are ascribed to this cause, and are noticed in the history of the case only as "tooth-ache." Presently, when the long continuance and progress of these changes have materially disorganized the periosteum, and qualified the nutrition of the bone, some accidental occurrence, (perhaps exposure to cold,) will determine an attack of acute inflammation: great tumefaction and severe pain arise; the new growth rapidly undergoes caries and dissolution; the soft parts as rapidly yield to ulceration or gangrene; the jaw, more or less completely, is at once struck with necrosis, and is seen lying in the midst of disorganized tissues, which pour out a profuse and insufferably fetid discharge.* From this point onwards, speaking surgically, the case is an ordinary one, viz. a sequestrum, larger or smaller, to be separated by the same processes of nature, or by the same operations of art, as a sequestrum produced under any other deleterious influence. I need hardly tell you that this second stage of the disease is one of great constitutional disturbance, under the first febrile influence of which, or under the subsequent exhaustive discharges, many patients die. It is likewise a period of extreme suffering.

From these general statements, you will be able to follow with facility and with interest, the history of two patients now in the hospital: one, (which I shall read first,) a very ques-

* It is stated that a large quantity of phosphorus is eliminated in this discharge—a point which I have had no means of verifying personally.—Vide Von Bibra u. Geist, op. cit., p. 338.

tionable case of the phosphorus-disease ; the other, a very complete and admirable illustration of it.

"L. G. F. H——, aged thirty-six, admitted into Abraham's ward, under Mr. Simon, on Oct. 23d, 1849, is a native and inhabitant of Nottingham ; is of middle height, thin, with dull, sallow complexion, and scanty black hair ; has worked at the business of a framework-knitter for the last twenty-six years, for sixteen hours daily, on an average ; habits, temperate and regular. At the time of his admission into the hospital, he had a deep ulceration at the right angle of the mouth, which had eaten away the substance of the lip, as far inwards as the median line, and downwards to the junction of lip and gum. The edges of this ulceration were peculiarly sharp and abrupt. Along the inner surface of the lip, the ulceration extended to the left commissure of the mouth, and at either side, obliquely, downwards to the gum. The left side of the jaw was covered with apparently healthy cicatrized gum, but had no teeth, and had lost its alveolar margin. On the right side, the last molar tooth remained firm and sound ; and one of the bicuspids also stood, with its inner root laid bare. The other teeth of this side were gone, as were likewise their alveoli. The remaining bone was partially scarred over. The under surface of the tongue was ulcerated down to the frænum, continuously with the ulceration of the lip. From these surfaces there was considerable discharge of healthy pus, unattended with fetor. In addition to this extensive sore, there was a single superficial circular ulceration, the size of a half-crown, situated on the cheek, over the angle of the jaw, not communicating with the interior of the mouth, and crusted over with a flat brown scab.

His account of himself was the following :

"Seventeen years ago, (1832,) he contracted primary syphilis, and had buboes in either groin ; never since that time had he suffered any venereal infection. Between 1835 and 1839, he had several attacks, described as erysipelas of the head ; but denies having had any distinct symptoms of secondary syphilis. Eight years ago, he suffered with some chronic cutaneous disease, for which he underwent medical treatment.

In June, 1846, the gums over the upper jaw, just under the nose, became very sore, red outside, and putrid within; the affection extended over the gums of the four front teeth, became gradually more and more painful, but was confined to the same space for about two months, when it gradually spread along the gums towards the back teeth, leaving the first part almost well. Presently, the throat became sore, and an abscess formed externally on each side, under the angle of the jaw; these were punctured, and a discharge proceeded from them for some weeks, when they healed on the outside, but began to discharge inside the throat; this lasted five months, and then ceased; but the gum, where the disease commenced, has ever since continued somewhat sore and red. During 1847 and 1848, he became subject to several symptoms indicating the presence of pulmonary tubercles; obstinate cough, followed and accompanied by hemoptysis, and, as the disease advanced, by purulent expectoration, diarrhea, and nocturnal perspiration. Through this period, he emaciated considerably, and since Sept., 1848, has been unable to follow his ordinary employment. Early in the present year, a friend recommended him to keep a piece of ginger-root constantly in his mouth, "to warm his chest," which he did, placing it in the front of his mouth, just within his lower lip, and carrying it, at other times, in his waistcoat-pocket. When occupying the latter situation, it lay with half a boxful of *lucifer-matches*, which he was in the habit of using occasionally, and which for this purpose he carried loose in his pocket. He continued to chew the ginger for about three weeks. A few days after commencing, he noticed that the ginger tasted disagreeably of phosphorus, but did not regard this, till, after using it for two weeks, he felt one day a gentle stinging in the gums, in front of the lower jaw, and over only a small space. The state of the teeth, at this time, was generally good, with the exception of the two bicuspids, on the right side of the lower jaw. Next day the stinging became more apparent, commencing from the right bicuspid, and day by day gradually grew more severe and more extensive, while the gums became red and swelled. In

April, two of the teeth, (canine and incisor,) the gums of which had been thus affected, became loose, and were extracted. Soon afterwards, he fancied that others were growing in their places, instead of which the two sockets appeared and fell out, as a decayed and friable piece of bone, as large as the last joint of the little finger. This exfoliation was accompanied by a very fetid discharge. After two or three weeks, two more of the teeth, (incisors,) were affected in the same way, were removed, and their alveoli followed; while the jaw generally got swelled and sore, discharging very foul matter. As the disease extended, the teeth continued to drop, two and two, with an interval of two or three weeks between, the disease proceeding first from the right bicuspids to the front, and then from before backward along the left side of the jaw, till all the teeth on that side were removed, with one exception; the last of them fell at the close of August. Then, from the first point of attack on the right side, the disease extended backward, the teeth becoming similarly affected, till all but two were removed, viz. the last molar, which continued sound and firm, and the bicuspid, which from the first had been decayed, and was now bare, down to the bottom of the inner fang, and rather loosened. During this period, (from April to August,) ulceration was proceeding on the gum, tongue, and inside of the lip; and about the middle of September had extended through the lip, in the form of the ulcer now observed there."

Now, to my view, gentlemen, this case presented the characters of constitutional syphilitic disease. I am not sure that I could have conveyed to another person the evidence that the man's general appearance gave to my mind, and which was confirmed by the deep sharp-cut chasm of his lip, and the circular crusted ulcer on his cheek. But taking his whole appearance and his whole history into account, I determined on treating him as though for syphilitic cachexia, and the result has justified that opinion. After a few days of treatment with nitric acid and quinine, during which, though he improved in general health, his ulcers remained stationary and painful, I commenced the exhibition of iodide of potass. The superficial sore on the cheek immediately began to cleanse itself, and to skin over; the deep ulcer began to granulate, and in the last few days has so nearly completed its cicatrization, that the man cannot now be prevailed on to remain any longer an inmate of the hospital.

The result of treatment, in this case, may perhaps contribute to fix the syphilitic nature of this patient's affection; it yielded to iodine with a readiness peculiar to that one class of diseases. And there are other reasons in favor of this view. He had suffered with an affection of the gums in the upper jaw three years before his lower jaw suffered, and though no necrosis occurred at that time, yet, not improbably, there was exposure of bone as a cause of the fetor then observed. Further, in the phosphorus disease, ulceration of the soft parts is unknown, except in immediate dependence on the separation of dead bone, and could not, on any pathological grounds, be supposed to continue for months after the last discharge of sequestrum, or to extend itself to surfaces so remote from the affected bone. It would not extend further in space, or longer in time, than an inflammatory infiltration could be traced; it would exist only as ulceration in an infiltrated part, and in this lip there was no thickening whatsoever. Still I must observe to you, that necrosis of the lower jaw, occurring in the manner described in this case, is not a frequent event in syphilis; and if we admit, as perhaps we may, that in this patient's case there has been continuous evidence of a cachectical state since his first syphilitic infection, we may yet allow the possibility of the phosphorus matches having acted concurrently with his cachexia, as a local determining cause for the disease.

You will notice, that in analysing this case, I have not alluded to the question of the sufficiency of the alleged cause for producing phosphorus-disease of the jaw, and I cannot pretend to decide whether the quantity of phosphorus introduced into the mouth by the patient's eccentric habit was enough to affect the bone. I have argued it on other grounds; but I may inform you that there are some reasons for supposing that a small quantity may suffice to produce very serious effects, and that Dr. Geist quotes an instance where exfoliation of several pieces of the lower jaw seems to have been induced in a little scrofulous girl, seven years of age, by her indulging herself, evening after evening, in the amusement of playing with lucifer matches, and watching their ignition in the dark.

The next case is more complete and satisfactory, and will enable you to form a good notion of the ordinary progress of symptoms in such cases.

"James Brewerton, aged forty-six, admitted under Mr. Simon, into Abraham's ward, July 31, 1849, with disease of the lower jaw, attributed by himself to the inhalation of phosphorus fumes. In giving an account of himself, he stated that till he was thirty-two years of age, he had pursued the various occupations of ironmonger, hard-store keeper, and trunk-maker, in this country and in the United States, and had uniformly enjoyed good health. He has never had syphilis, nor undergone mercurial treatment. Once he had gonorrhea, which was cured by copaiba. He began to make lucifer-matches, on his own account, in 1835, using a composition of chlorate of potash and antimony, and did not begin to use phosphorus till he commenced the manufacture of 'congreves,' in 1837. At that period he was an active, healthy man, thirty-four years old. The manner of making 'congreves' was then a secret, and he used to mix the ingredients by himself, in a private room, to which the work-people, who dipped and packed, were not admitted. The mixing always occupied half an hour daily, but in addition, he spent much time in making various experiments, to ascertain the best proportion of ingredients, &c., so that he was much exposed to phosphorus fumes, in, usually, an ill-ventilated room. He never used arsenic. After he had mixed and given out the materials, he was constantly in the apartments where the dipping and drying of the matches were carried on. The only burning of phosphorus was such as arose from accidental explosions of small quantities of matches.

"To the best of his recollection, he had no decayed teeth in 1837, when he first became exposed to the fumes of phosphorus. From 1837 till 1845, the patient continued actively engaged in his trade.* During the years 1846–7, and –8, he left off making matches, and made instead, 'fusees,' for lighting cigars; the materials being the same as for congreves, but their employment, perhaps, less hurtful, as no heat is employed in mixing them. It was while making fusees that he first began to be troubled with carious teeth; the three molars on the left side of the lower jaw being so painful that he was obliged to masticate on the right side exclusively. Afterwards, the teeth on the right side pained him, and he was forced to employ the left ones again. At Christmas, 1848, he resumed the manufacture of congreves, moving, for this purpose, from his former residence to Nottingham.†

"In the latter town, he occupied larger and better-aired premises than had previously been used there, and mixed all the composition with his own hands. The work went on regularly, except now and then, when they had to wait for want of materials from London. On such occasions, there was a pause for a week or so.

* While detailing his own case, he mentioned, that after he had been about four years congreve-match making, two boys, who had been during that time employed by him, and in good health, went to another similar manufactory, and, (he was told,) were attacked with disease of the jaws, and died.

† The circumstance which occasioned his migration is worth mentioning, as it gives an additional illustration of the disease. A friend of his, who had been constantly employed in congreve-match making, at Derby and Nottingham, for six years, was incapacitated from work by his jawbones becoming diseased. He, (like Brewerton,) had always mixed his materials in private, for the sake of secrecy, and in a close, small room, where much of the manufacturing was afterwards carried on—so that he was even more exposed to inhale the phosphorus fumes than Brewerton had been. This person had been under the care of Dr. Taylor, of Nottingham, in the summer of 1848, and lost at that time a large portion of the superior maxillary bones. Dr. Taylor's kindness has contributed these specimens to our museum. The same disease afterwards attacked the right half of this man's lower jaw. The second attack obliged him to desist from work, and invite the assistance of Brewerton, who hereupon moved from London to Nottingham.

"About the end of May, Brewerton lay for some time on the bank of the river, waiting to see a boat-race. The ground was damp, from rain having fallen two or three days before; and when, after a few days, he found a swelling about the left side of the lower jaw, accompanied with pains in the bone, extending to different parts of the head and face, he attributed the symptoms to his having caught cold. He consulted Dr. Taylor, who extracted two loose and decayed teeth, (left molars,) and the patient himself took out another. All these teeth were rotted away at the crowns, but their fangs were entire.

"At the time of his admission, at the end of July, his condition was as follows: Three remaining incisors quite loose; entire gum on left side swollen and spongy; fetid pus escaping, on the slightest pressure, from the previous site of the extracted teeth; a probe passed into these openings strikes on bare bone; over these parts there was considerable exterior swelling; pulse quick and feeble; general strength impaired; face puffy and very pallid; appetite good, but inability to take solid food, owing to condition of gums; sleep disturbed by pains in the jaw; and complaint also of general articular pains.

"On the 5th August, the remaining incisors were extracted; and on the 15th, the dens sapientiæ of the affected side. On the 22d, the right canine tooth came out, and the bicuspids were found to be loose; and pain and swelling increased on this half of the face, speedily bringing it into the same condition as the half first affected.

"Meanwhile the ulceration of the gum over the diseased alveoli advanced; and as the several openings became confluent, the alveolar margin of the bone became exposed. This commencement once made, the soft parts rapidly retracted from the surface of the bone, and the denudation of the latter became every day more complete. Its exposure was attended with a copious and most fetid suppuration.

"From then till now, the only difference that has occurred in the patient's state, has consisted in the gradually advancing isolation of the bone. His general health has also undergone considerable depression; he keeps his bed entirely; is very pale and feeble : has an exceedingly weak pulse; occasionally gets a little chilly or feverish; and has, from time to time, some disposition to looseness of the bowels; the appetite, fortunately, keeps good."

At the present time, as you look at the man, you see that the lower half of his face is considerably enlarged and hardened by deposit round the inferior maxilla; and you may observe that this deposit, (which extends half way up the ascending ramus on one side, and still higher on the other,) increases in thickness as it approaches the lower margin of the bone. Part of this swelling depends on inflammatory infiltration into the areolar tissue and muscles; part on the chronic osseous incrustation which I have described to you, and which is now undergoing caries. Within these swollen parts, as you open the mouth, you see and feel the whole body of the jaw uncovered, necrosed and discolored. You see that the soft parts have detached themselves, and shrunken from the bone, both in front and behind, so that it lies bare as far back as the junction of its body and rami. Beyond this line, the soft parts still cover the bone, but apparently are unattached to it through a still further extent of surface. The whole body of the lower jaw, with probably a considerable share of each ramus, has to separate itself, and be cast off as a sequestrum.

Now, gentlemen, with respect to the treatment of this case in its present stage, there are two or three points to which I must direct your attention. First, you may notice that I leave the jaw pretty much to itself. I give the patient the means of cleansing his mouth frequently with deodorizing and astringent lotions, sometimes of myrrh, sometimes of chloride of lime; (I say *lotions*, because latterly he has been unable to effect his purpose by using them as gargles, and has used them by means of pieces of sponge attached to a wooden holder like a pencil-stick.) This is an important part of the treatment, because of the extreme fetor of the discharge, and the disgust and sickness which it occasions to the unfortunate patient, as well as to other persons. As regards the separation of the jaw, I am unwilling to adopt any active surgical interference. In this respect, I look on the case as I should look on one of necrosis of half the shaft of the femur, or tibia. I know that nature is working on at the process of separation, and I know that if the man's strength holds out, she will complete that process in a neater and less destructive manner than I could effect it by surgical interference. In other severe cases of necrosis, such as I have just instanced, we are not very often driven to amputation; we are generally enabled, by judicious general treatment, to sustain the vital powers of the patient till nature has divided the dead parts from the living, and permitted us to extract the former, as a sequestrum, without having recourse to any mutilative operation. So I hope to do here: I hope to support the patient till his necrosed jaw detaches itself, and then to draw it out of his mouth. Nature, in this event, will gradually have accommodated the soft parts to the change, and the subsequent deformity will be far less than if I should now disarticulate the jaw. Further, I take into account that the latter operation, under the most favorable circumstances, is a serious one; and that in a case like the present, where the soft parts are extensively, and, to a certain extent, idiopathically diseased, it would be especially so; for if erysipelas should arise after the operation, (a chance always to be contemplated as possible in hospital practice,) all these soft parts would infallibly slough. The man's general health, too, is not such as to invite any surgical operation which involves the extensive division of diseased and infiltrated tissues. Therefore I am anxious to avoid any operative interference with the natural processes of separation; but if, contrary to my hope, the man's health should give way—if his appetite should fail—if he should emaciate much more—if hectic fever should arise and continue—if diarrhea, (which has occasionally troubled him,) should assume a serious complexion—if extensive perforation of the skin should be impending—if deglutition should be interfered with—I might, under any of these circumstances, be driven from my position of *médicine expectante*, and be compelled to expose and disarticulate the jaw.

Meanwhile, you will observe that I am careful to give the patient as much support as diet and physic will convey. His ghastly, anæmic look made me, on first seeing him, think that he would bear iron with advantage; but, to my surprise, he has shown himself quite intolerant of the remedy. I have tried it in three forms—citrate, sulphate and sesquichloride; and all these have so purged him, that I have been obliged to discontinue their use, and to substitute for them the sulphate of quinine. This drug has probably been useful, though less so than steel would have been if he could have borne it.

When his pain has been very severe, I have allowed him a night dose of hydrochlorate of morphia; but have urged him to refrain as long as he can from seeking this narcotic, lest its employment should impair his appetite.

When his bowels have shown a disposition to run, I have checked them by doses of catechu, which I have combined with the compound tincture of cinchona.

You will notice, too, that, in respect of his diet, I do not leave him to the chance of mumbling hard victuals. I allow him extra meat, and have this reduced to a purée, or mash, by pounding in a mortar, so that he may bolt it without further mastication. He likewise has other fluid or semi-fluid nourishment; eggs, strong beef-tea, and the like; and is allowed a very liberal quantum of wine, occasionally with other stimulants, which I increase from time to time, as the condition of his general strength indicates a necessity. I detail these points of treatment to you, because they require care and vigilance, and because the issue of the case depends on them very much more than on the pharmacopœia.

I have not detained you with any remarks on the management of the earlier stage of the disease, anterior to necrosis, because I have not at present any opportunity of illustrating that stage to you. I will merely remind you, that so soon as the second or inflammatory stage of the disease has thoroughly set in, the bone seems in every case to be irrecoverably doomed to necrosis; and I would therefore recommend you, in the event of your being called to a case at the transition-period between the two stages, when hypertrophy is passing into inflammation, to adopt, without hesitation, the most active measures for relief of the periosteum and bone. Leeches and general antiphlogistic treatment may do good; but the consideration of the pathology of this disease, together with the analogy of other periosteal affections, leads me to believe that the only real chance of doing good would lie in still more energetic measures; and I would recommend you, in any such instance, to make, with your scalpel, free vertical incisions through the gum, wherever tenderness and swelling exist; extending your line of cut upwards in the upper jaw, or downwards in the lower, as far as the structure of the parts will allow, bringing your incisions as near together as circumstances may require, and in every point carrying them clearly down to the bone, so as to afford the utmost relief and relaxation to the overloaded and tense periosteum. I believe that this method of procedure, (somewhat analogous to Mr. Tyrrell's for the relief of the cornea in gonorrheal ophthalmia,) would be the nearest approach to an effective one for checking the inflammatory stage of the disease, before it has reached an intensity which must inevitably destroy the jaw.

